# Metagenomics and metabolomics integrated to explore the protective mechanisms of Mongolian medicine Zadi-5 in myocardial ischemic model rats

**DOI:** 10.3389/fmicb.2025.1677322

**Published:** 2026-02-09

**Authors:** Riga Wu, Wen Zu, Lisi Wei, Rihan Wu, Si Su, Ruhan A, Tengarile A, Nirile E, Hua Li, Rilebagen Hu, Li Li

**Affiliations:** 1 Mongolian Medicine College, Inner Mongolia Medical University, Hohhot, China; 2 Pharmacy College, Inner Mongolia Medical University, Hohhot, China; 3 Basic Medical College, Inner Mongolia Medical University, Hohhot, China

**Keywords:** gut microbiota, metabolism, Mongolian medicine, myocardial ischemia, Zadi-5 pill

## Abstract

**Background:**

Myocardial ischemia (MI) is a pathological state of abnormal energy metabolism caused by insufficient blood and oxygen supply to the coronary arteries. The “gut-heart axis” theory plays an important role in myocardial ischemia occurrence, mechanism, prevention, and cure. Traditional Mongolian medicine posits that “internal diseases originate from gastrointestinal dysfunction,” linking the intestine, a key component of the digestive system, to physiological and pathological changes in the heart. Furthermore, the traditional Mongolian clinical treatment of cardiovascular diseases includes guidelines for digestive system function corresponding to the modern concept of the gut-heart axis. Accordingly, Zadi-5, a traditional Mongolian medicine, has been used for over 200 years to prevent and treat cardiovascular diseases. However, the mechanism by which the gut microbiota and metabolism are regulated to protect an ischemic heart is unclear.

**Aim:**

This study aimed to investigate the potential mechanism by which Zadi-5, through its interaction with the gut microbiota and metabolic pathways, alleviates myocardial ischemic injury induced by a high-fat diet and isoproterenol (ISO).

**Methods:**

Sprague-Dawley rats were divided into control, model, Zadi-5 high-dose, and Zadi-5 low-dose groups. All groups, except the control group, were fed a high-fat diet for 4 weeks. Subsequently, all animals received subcutaneous injections of 4 mg/kg ISO daily for 3 days to induce a myocardial infarction (MI) rat model. The pharmacological effects of Zadi-5 on MI were assessed using electrocardiography (ECG), hematoxylin-eosin (HE) staining of myocardial tissue, and serum levels of cardiac troponin T (cTn-T), creatine kinase-MB (CK-MB), lactate dehydrogenase (LDH), total cholesterol (TC), triglycerides (TG), low-density lipoprotein cholesterol (LDL-C), and high-density lipoprotein cholesterol (HDL-C). Furthermore, fecal metagenomics and serum untargeted metabolomics were performed to investigate the protective mechanisms of Zadi-5 against MI. Finally, MetOrigin was used to analyze the correlation between key metabolic pathways and the gut microbiota to elucidate the mechanism by which Zadi-5 protects against myocardial ischemia.

**Results:**

First, the MI rat model was successfully established by ISO, and Zadi-5 significantly preserved MI injury, according to ECG recording, index of TC, TG, LDL-C, cTn-T, LDH, CK-MB, and histopathology results. Second, Zadi-5 regulates gut microbiota diversity and abundance, as well as glutamine and glutamate metabolism. The mechanism is related to the gut microbiota phyla *Actinobacteria, Firmicutes, Bacteroidetes*, and *Proteobacteroidetes*, and classes *Gammaproteobacteria, Betaproteobacteria, Bacteroidia, Actinomycetes, Clostridia, and Bacilli*. Zadi-5 also regulates L-glutamic acid, L-glutamine, ornithine, and oxaceprol metabolisms.

**Conclusion:**

Zadi-5 exerts cardioprotective effects in MI rats by improving dysbiosis of the gut microbiota and regulating the glutamate–glutamine metabolism pathway. This may represent only one of the complicated protective mechanisms of Zadi-5 against MI. The cardioprotective mechanisms of Zadi-5 will be explored at the molecular and cellular levels.

## 1 Introduction

Cardiovascular disease (CVD) is a leading cause of morbidity and mortality worldwide (Goldsborough et al., 2023). Specifically, more than 30% of deaths worldwide are caused by coronary heart disease (CHD) (Mokhtari et al., 2022), which often manifests as ischemia-induced myocardial injury. The pathological changes of myocardial ischemia (MI) are mainly attributed to vascular lumen stenosis or obstruction due to vascular epithelial injury, metabolism disorder, platelet aggregation, and several other phenomena, including lipid metabolic disorders (Ko and Kim, 2020). The pathological mechanism of MI-induced CVD is complicated, and several hypotheses have been proposed (Guo et al., 2022). For example, oxidative stress, apoptosis, inflammation, and autophagy participate in myocardial ischemia, affecting myocardial structure and function, which ultimately leads to heart failure (Zhang et al., 2023).

**Figure 1:**
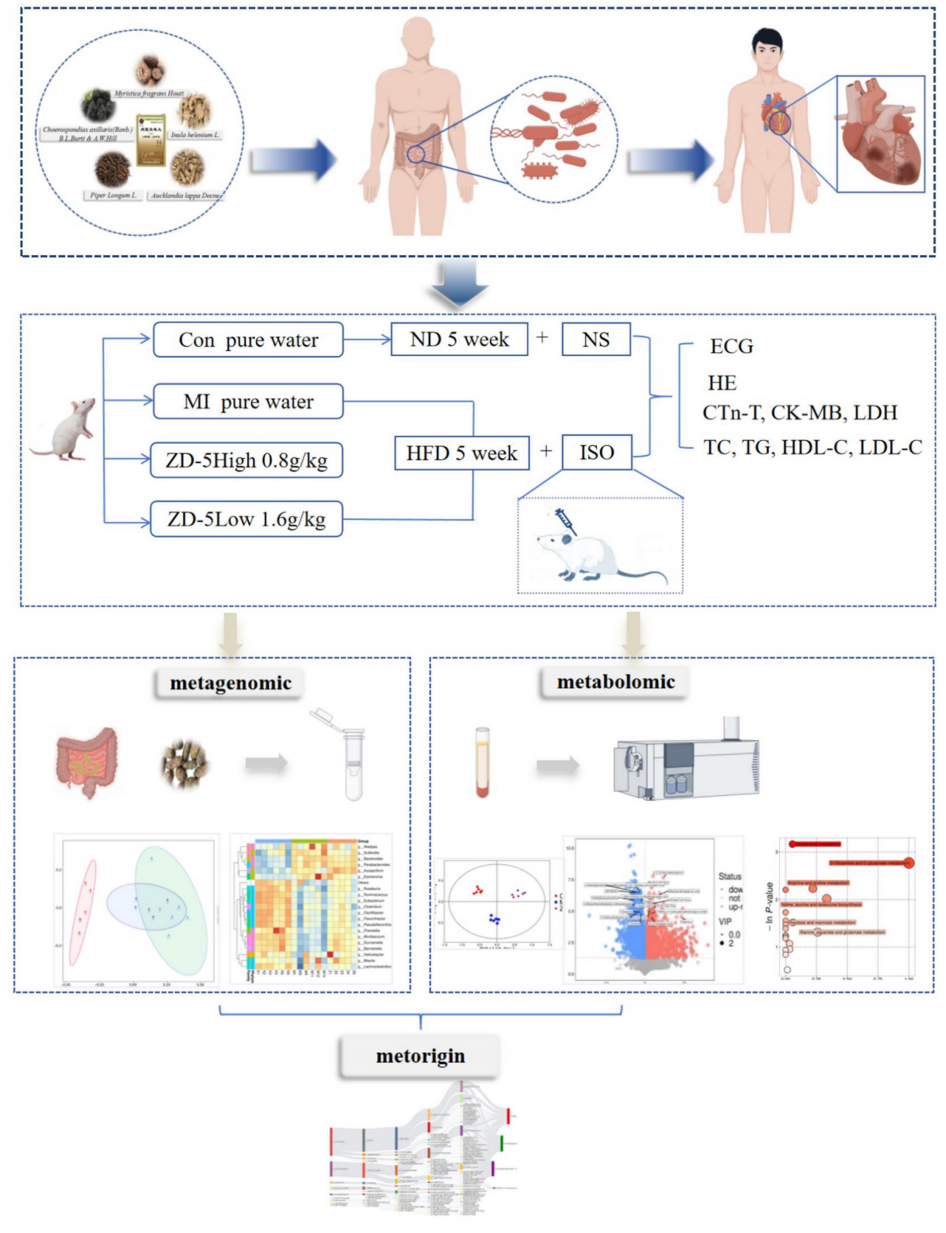
Graphical summary of the study design. CON, control group; MI, myocardial ischemia group; ZD-5, Zadi-5; HFD, high-fat diet; ND, normal diet; ECG, electrocardiogram; HE, hematoxylin-eosin staining.

Emerging research has highlighted the gut-heart axis as a significant contributor to cardiovascular outcomes. This axis represents a dynamic, bidirectional network in which homeostasis of the gut microbiota plays a pivotal role in metabolic balance in the host (Chen et al., 2024; Jain et al., 2024; Pitt and Diez, 2024). Gut microbiota is widely considered important for understanding CVD occurrence and development. The gut microbiota interferes with cardiac function through metabolites that undergo intestinal absorption and enter the systemic circulation. These metabolites induce intrinsic signaling cross-talks in the gut microbiota-heart axis, contributing to CVD development (Datta et al., 2024). Such an altered microbiota is associated with cardiometabolic diseases, producing disease-causing and disease-modifying metabolites (Nageswaran et al., 2025). A recent meta-analysis combining 15 studies observed that *Bacteroidetes* and *Proteobacteria* are increased in CVD (Kondapalli et al., 2025). Thus, exploring gut microbiota regulation and cardioprotective functional drugs is important for clinical treatment. Mongolian medicine suggested that “internal disease originates from the gastrointestinal dysfunction” and “there is an inseparable physiological and pathological relationship between the heart and the intestine.” Thus, protecting and restoring the digestive system is particularly important in CVD treatment. This is similar to the concept of the heart-gut axis. The formula of Zadi-5, a classical Mongolian medicine for treating CVD, comprises five natural plants: *Myristica fragrans Houtt, Inula helenium L., Aucklandia lappa Decne., Choerospondias axillaris (Roxb.) Burtt et Hill*, and *Piper longum L*. Zadi-5 has been used for palpitation, arrhythmia, angina, myocardial infarction, and MI-related diseases for >200 years. The pharmacological activities of *Myristica fragrans Houtt* exert cardiovascular-protective, antioxidant, and anti-inflammatory effects (Ashokkumar et al., 2022). Furthermore, *Aucklandiae Radix* could mitigate gastric ulcers by reducing tumor necrosis factor-α, interleukin-1β, and myeloperoxidase levels, while improving gastric histopathological features *in vivo* (Feng et al., 2023). *Inula helenium L* contains antioxidant and antibacterial constituents such as secoiridoid glycosides, phenolic acids, and flavonoids (Buza et al., 2022). Additionally, *Piper longum L*. is characterized by antimicrobial, anti-inflammatory, and anti-hyperlipidemic activities and is used in Traditional Chinese Medicine for pain relief, coronary heart disease, stroke, stomach disease, and other medical conditions (Tiwari et al., 2023; Biswas et al., 2022). Zadi-5 improves digestive function and exerts cardioprotective effects. Our previous research demonstrated that Zadi-5 decreases the levels of myocardial enzymes, such as cardiac troponin (CTn-T), creatine kinase myocardial band (CK-MB), and lactate dehydrogenase (LDH); alleviates pathological and morphological changes in cardiomyocytes; and exerts anti-apoptotic effects through the PI3K/AKT and Bcl-2/Bax signaling pathways (Wei et al., 2021; Wuri et al., 2024). This study aimed to explore the complicated myocardium-protecting mechanism of Zadi-5 based on previous research on Zadi-5 and a holistic view of the traditional Mongolian medicine theory of the gut-heart association, connected to the modern gut-heart axis concept, mainly dependent on fecal metagenomics, serum untargeted metabolomics, and Metorigin analysis (Figure 1).

## 2 Materials and methods

### 2.1 Chemicals and reagents

Methanol [CNW Technologies, China; Chemical Abstracts Service [CAS] No. 67-56-1], acetonitrile (CNW Technologies, China; CAS No. 75-05-8), ammonium acetate (Sigma-Aldrich, USA; CAS No. 631-61-8), ammonium hydroxide (Fisher Chemical, USA; CAS No. 1336-21-6), isoflurane (Shan Dong An Te Animal Technology Co., Ltd., China; CAS No. 26675-46-7), cTnT, CK-MB, LDH, total cholesterol (TC), low-density lipoprotein cholesterol (LDL-C), high-density lipoprotein-cholesterol (HDL-C), triglyceride (TG) detection kit (Mai ke Biological Co., Ltd, China), isoproterenol (ISO) (Sigma-Aldrich, USA), and HE staining system (Leica Biosystems, Germany; Catalog No. 3801655) were used in this study.

### 2.2 Zadi-5 preparation

Zadi-5 is a traditional Mongolian medicinal formulation listed in the Mongolian Medicine Product Standards. It is composed of five herbs: *Myristica fragrans Houtt*. (31.25%), *Inula helenium L* (25%), *Aucklandia lappa Decne*. (25%)*, Choerospondias axillaris (Roxb.) Burtt et Hill* (15.63%), and *Piper longum L* (3.12%). The plant names were checked through the http://www.theplantlist.org website. All herbs used in this study were obtained from Hebei Jixintang Pharmaceutical Co., Ltd. (Hebei, China), Anguo Runde Pharmaceutical Co., Ltd. (Hebei, China), and Anguo Jiuwang Pharmaceuticals Co., Ltd. (Hebei, China). The quality control data and relevant marker constituents are listed in Supplementary material 1. Ultra-high-performance liquid chromatography-triple/time-of-flight mass spectrometry (UHPLC-Q-TOF-MS/MS) samples were prepared using the following protocol. The herbs were pulverized and mixed in an appropriate ratio, followed by the addition of 85% ethanol. Using a Soxhlet extractor, the mixture was extracted for 2 h to obtain 2 mL of the sample for analysis. Low and high doses of Zadi-5 were prepared as 0.8 and 1.6 g/ml solutions (in ultrapure water), respectively, for animal experiment preparation.

### 2.3 Identification of the Zadi-5 components

Chromatography was performed using a system from Waters Corporation (USA). The extracted Zadi-5 solution to be tested was separated using an ACUITY UPLC^®^ BEH C18 column (2.1 × 100 mm, 1.7 μm), followed by electrospray ionization (ESI) with detection in ESI-positive and -negative modes. The instrument was controlled by the Unifi 1.8.0 package (Waters Corporation, USA; Supplementary material 2).

### 2.4 Animal experiments

#### 2.4.1 Grouping and intervention

Specific pathogen-free (SPF) Sprague-Dawley (SD) rats were randomly divided into control (Con), MI model (MI), Zadi-5 high dose, and Zadi-5 low dose groups (the doses were determined by human doses). All groups were fed a high-fat diet (HFD), except for the Con group, which received a normal diet. Rats were gavaged with high (1.6 g/kg) and low (0.8 g/kg) Zadi-5 doses at a value of 1 mL/kg at 9:00 am for 4 weeks in both the Zadi-5 high- and low-dose groups. Then, the MI, Zadi-5 high, and Zadi-5 low groups received a subcutaneous injection of 4 mg/kg ISO in the neck every day for 3 days (Alshehri, 2022). The MI model was considered successfully established, provided there was elevation or reduction in the ST segment (J point shift down, T wave disappeared), a high or absent T wave, or a pathological Q wave.

#### 2.4.2 Sample collection

After 3 h of model establishment, an electrocardiogram (ECG) of the rats was recorded after anesthesia induction. Laparotomy was conducted to collect abdominal aortic blood to analyze CK-MB, cTn-T, LDH, TC, TG, LDL-C, and HDL-C levels. Additionally, the heart was stained with hematoxylin and eosin (HE).

#### 2.4.3 16SrDNA metagenomic sequencing and analysis of fecal samples

The Fecal Genome DNA Extraction Kit (AU46111-96, BioTeke, China) was used to extract total DNA from feces. Metagenome libraries were sequenced using an Illumina NovaSeq 6000 platform with PE150. Next, the sliding-window algorithm method was used to trim low-quality reads (quality scores < 20) and short reads (< 100 bp). The remaining reads are subjected to *de novo* assembly for each sample using MEGAHIT (v1.2.9). We retained contig sequences with lengths greater than 500 bp for CDS (Coding Region) prediction using MetaGeneMark. Clustering was performed with an identity of 95% and coverage of 90% using CD-HIT (v4.6.1) to obtain unigenes. A taxonomic assessment of the microbiota was performed using DIAMOND (v 0.9.14), based on the NR database. Microbial functions were assigned using KEGG.

#### 2.4.4 UHPLC-OE-MS non-target metabolomic analysis of serum

First, 100 μL of the serum sample was mixed with 400 μL of the extraction solution (MeOH: ACN, 1:1, *v/v*). The mixed solutions were vortexed, sonicated, and incubated for 1 h at −40 °C to precipitate proteins. Afterward, the samples were centrifuged, and the supernatant was transferred to a fresh glass vial for analysis. The QC sample was prepared by mixing an equal volume of supernatant from each sample. LC-MS/MS analyses were performed using a UHPLC system (Vanquish, Thermo Fisher Scientific) with a Waters ACQUITY UPLC BEH Amide (2.1 mm × 50 mm, 1.7 μm) coupled to an Orbitrap Exploris 120 mass spectrometer (Orbitrap MS, Thermo Fisher Scientific). The mobile phase comprised 25 mmol/L ammonium acetate and 25 ammonia hydroxide in water (pH = 9.75) (A) and acetonitrile (B). The analysis was performed with the following elution gradient: 0–0.5 min, 95% B; 0.5–7.0 min, 95%−65% B; 7.0–8.0 min, 65%−40% B; 8.0–9.0 min, 40% B; 9.0–9.1 min, 40%−95% B; 9.1–12.0 min, 95% B. The flow rate was 0.5 mL/min. The column temperature was 30 °C. The auto-sampler temperature was 4 °C, and the injection volume was 2 μL. The QE HFX mass spectrometer was used for its ability to acquire MS/MS spectra in the information-dependent acquisition (IDA) mode in the control of the acquisition software (Xcalibur, Thermo). The ESI source conditions were set as the sheath gas flow rate of 30 Arb, aux gas flow rate of 25 Arb, capillary temperature of 350 °C, full MS resolution of 60,000, MS/MS resolution of 7,500, collision energy of 10/30/60 in the NCE mode, and spray voltage of 3.6 kV (positive) or 3.2 kV (negative). Finally, SCIEX OS software (AB SCIEX, USA) and the HMDB database (https://hmdb.ca/) were used to identify and validate them. Furthermore, we performed traceability analysis of differential metabolites using MetOrigin (http://metorigin.met-bioinformatics.cn/), in which origin, function, and Sankey network analyses were performed using the simple MetOrigin analysis mode available on their website.

### 2.5 Statistical analysis

All results are presented as mean ± standard deviation. Using the Statistical Package for Social Sciences (SPSS) for statistical analysis, statistical significance was calculated using the two-tailed Student's *t*-test when comparing two conditions and the ANOVA LSD *post-hoc* test or Tamhane's T2 when comparing more than two conditions. A *P*-value < 0.05 indicated statistical significance.

## 3 Results

### 3.1 Zadi-5 protects the cardiac structure and function in MI rats

**Figure 2:**
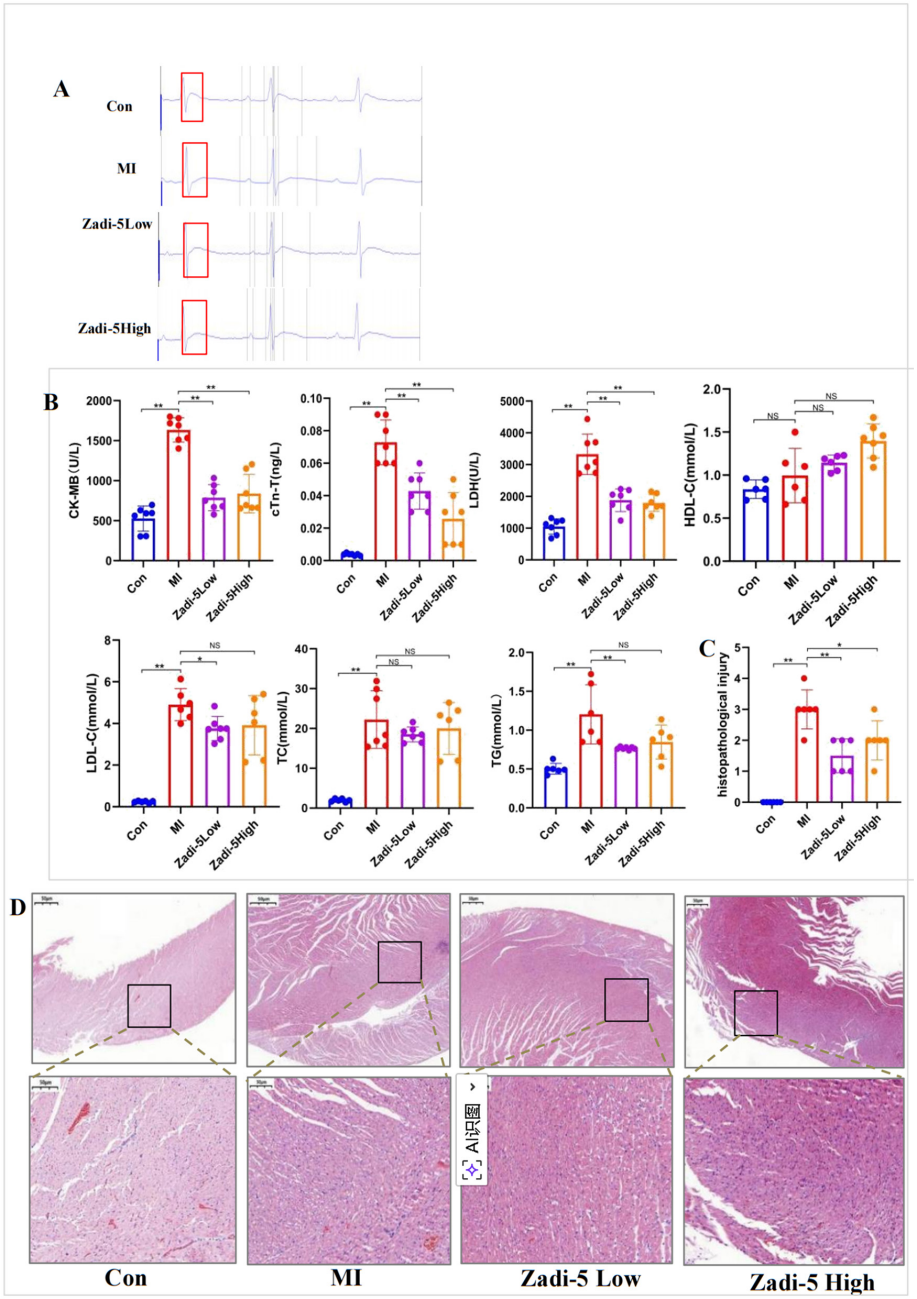
Zadi-5 regulates the myocardial function and structure in MI rats. **(A)** ECG records of each group. **(B)** Myocardial function and hyperlipemia-related indices. **(C)** Myocardial histopathology infarct size. **(D)** Myocardial histopathological changes. *P < 0.05, **P < 0.01, NS represents no statistical significance.

In this study, ECG showed a J-point shift, and the downward T wave disappeared in MI model rats, confirming successful MI rat model establishment, whereas ECG was relatively normal in the Zadi-5 high and low groups (Figure 2A). Regarding blood lipid lesions, TC, TG, LDL-C, and HDL-C levels in the MI group were higher compared to those in the Con group. TC, TG, and LDL-C levels in the Zadi-5 high and low groups were reduced, whereas HDL-C levels were increased compared to those in the MI group. On the other hand, serum cTn-T, LDH, and CK-MB levels in the MI group were significantly higher compared to those in the Con group (*P* < 0.01). The Zadi-5 high and low groups showed significant reductions in these levels compared to the Con group (*P* < 0.01) (Figure 2B). Myocardial histopathological analysis indicated that the cardiac tissue in the Con group was neatly arranged, with no damage to the fibers or inflammation. On the other hand, the cardiac tissue arrangement in the MI group was significantly disordered, with signs of severe fibrosis, inflammatory cells, and partial necrosis. Compared to the MI group, the Zadi-5 high and low groups showed significant improvements, in which the degree and scope of cell necrosis were significantly reduced with minor populations of inflammatory cells. However, the Zadi-5 low group showed no significant changes (Figures 2C, D).

### 3.2 Zadi-5 regulates the intestinal microbiota in MI rats

#### 3.2.1 Quality control of sample metagenomic sequencing data

**Figure 3:**
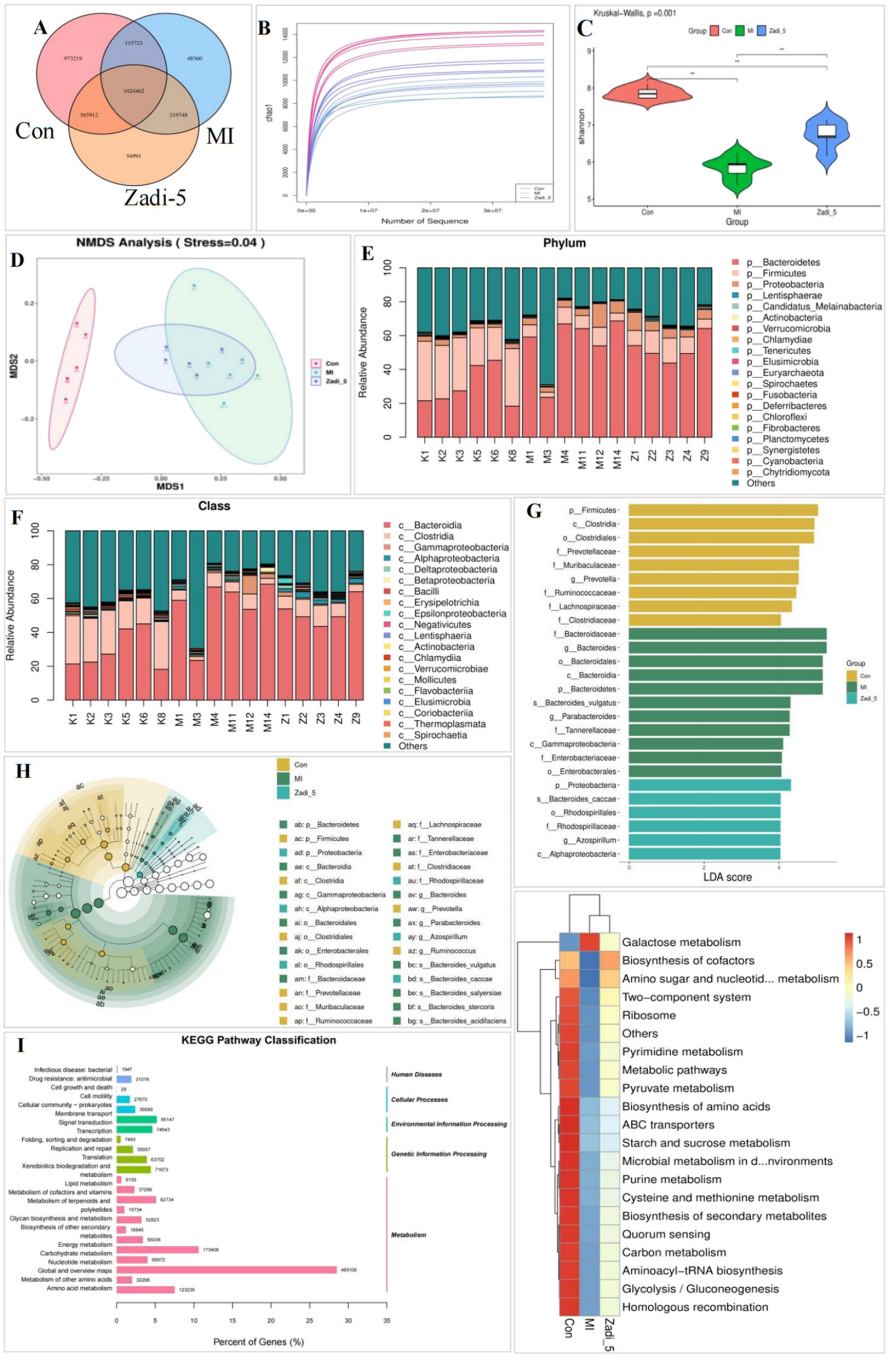
Metagenomic analysis. **(A)** Venn diagram of the unigenes in the Con, MI, and Zadi-5 groups. **(B)** Rarefaction curve of α diversity. **(C)** α diversity of the Shannon index. **(D)** β Diversity NMDS analysis of samples in Con, MI, and Zadi-5 groups. **(E)** Intestinal microbiota abundance of the phylum. **(F)** Intestinal microbiota abundance of the class. **(G)** Histogram of LDA analysis. When species with an LDA Score>4 were statistically different, the length of the histogram (LDA Score) represented the impact size of the different species. **(H)** The distribution difference of the microbiota was analyzed using LEfSe. **(I)** KEGG pathway analysis at levels 1 and 2. **(J)** KEGG pathway heat-map analysis at level 3.

Metagenomic sequencing of the intestinal microbiota was performed using colon fecal samples from each group to observe changes in the intestinal microbiota species in MI rats before and after Zadi-5 treatment. A Venn diagram was used to illustrate the common and unique unigene counts in the Zadi-5 groups compared to those in the MI and Con groups (Figure 3A). Analysis of the rarefaction curves (Figure 3B) revealed that all experimental groups showed sufficient sequencing depth, similar richness, and a high detection rate of microorganisms, meeting the standards of sequencing and database analysis.

#### 3.2.2 Intestinal microbiota diversity analysis

Alpha diversity reflects the richness and evenness of species, referring to the diversity within a specific environment or ecosystem via indices such as the Shannon index. The alpha diversity of the MI group was lower compared to that of the Con group. Furthermore, the alpha diversities of the Zadi-5 group increased to varying degrees compared to the MI group. Hence, HFD- and ISO-induced MI models could significantly reduce the diversity of the intestinal microbiota in rats. Additionally, Zadi-5 administration significantly increased the diversity of the intestinal microbiota in rats with MI (Figure 3C). Beta diversity contributes to the overall diversity or biological heterogeneity of a certain environmental community and refers to species differences between different environmental communities. Beta diversity analysis usually starts by calculating the distance matrix between environmental samples, which contains the distance between any two samples, and mainly observes the differences between samples through Nonmetric Multidimensional Scaling (NMDS). Different colors represent different groups in the results. The distances among the points represent different degrees of samples, and stress < 0.05 indicates good representative analysis results. The NMDS analysis results showed that the clouds of the Con and MI groups were separated, indicating that MI induction altered the composition of the intestinal microbiota in rats. The MI and Zadi-5 group distributions were similar, but most of the samples were discrete. Therefore, there were some common and unique intestinal microbiota compositions in these groups (Figure 3D).

#### 3.2.3 Changes in intestinal microbiota abundance

According to metagenomic analyses, unigenes can be categorized into phyla, classes, orders, families, genera, and species. The intestinal microbiota structure in each group displayed significant alterations. The heatmap depicting the abundance of unigenes across taxa utilized clustering and color gradients to illustrate the similarities and differences among multiple groups at various taxonomic levels. The relative abundance of phyla and class taxa of the intestinal microbiota composition in each group is shown. At the phylum level, *Firmicutes* and *Bacteroidetes* were the dominant microbiota in the rats (Figure 3E). In the class taxon, *Bacteroidia* and *Clostridia* were the dominant ones (Figure 3F). Linear discriminant analysis effect size (LEfSe) was employed to identify and resolve statistically significant differences in the marker flora (*P* < 0.05, LDA score >4.0) between the groups (Figure 3G). The LEfSe analysis revealed 26 marker microbiota. In the Con group, the dominant microbiota were *Firmicutes, Clostridia*, and *Clostridiales*. The MI group contained *Bacteroidaceae, Bacteroides, Bacteroidales, Bacteroides, and Bacteroidetes species*. In the Zadi-5 group, the dominant microbiota comprised *Proteobacteria, Bacteroides-caccae, Rhodospirillales, Rhodospirillaceae, Azospirillum*, and *Alphaproteobacteria*. Evolutionary branching trees within a clade illustrate the classification levels from phylum to species. The yellow nodes represent the significant microbial groups in group Con, whereas the green nodes signify the importance of microbial groups in group MI. The blue nodes represent significant microbial groups in the group Zadi-5. Additionally, yellow nodes indicate species that did not show significant differences (Figure 3H). The evolutionary relationships of the markers are as follows: *Firmicutes*-*Clostridia*-*Clostridiales*-*Clostridiaceae, Ruminococcus*, and *Ruminococcaceae*-*Lachnospiraceae*. *Bacteroidetes-Bacteroidia-Bacteroidales*. *Proteobacteria-Gammaproteobacteria, Alphaproteobacteria-Enterobacterales, Rhodospirillales-Enterobacteriaceae, Rhodospirillaceae*.

#### 3.2.4 KEGG pathway analysis of the intestinal microbiota DNA

According to the different unigenes of the gene functional KEGG pathway analysis, metabolism was predominantly enriched at level 1. At level 2, 12 pathways were highly enriched, including lipid, amino acid, energy, and carbohydrate metabolisms (Figure 3I). At level 3, the KEGG pathway definition was enriched in each group of specific metabolic pathway changes, as displayed in the heat map. In the top 20 pathways, MI injury decreased the enrichment of each metabolic pathway, while Zadi-5 could regulate the changes in the enrichment, except for galactose metabolism. In MI-injured rats, metabolic pathway disorder was the crucial mechanism of intestinal microbiota distribution and dysfunction, as well as the pharmacological mechanism of Zadi-5 in MI injury (Figure 3J).

### 3.3 Zadi-5 regulates metabolomics in MI rats

#### 3.3.1 Zadi-5 altered the blood serum metabolites in MI rats

**Figure 4:**
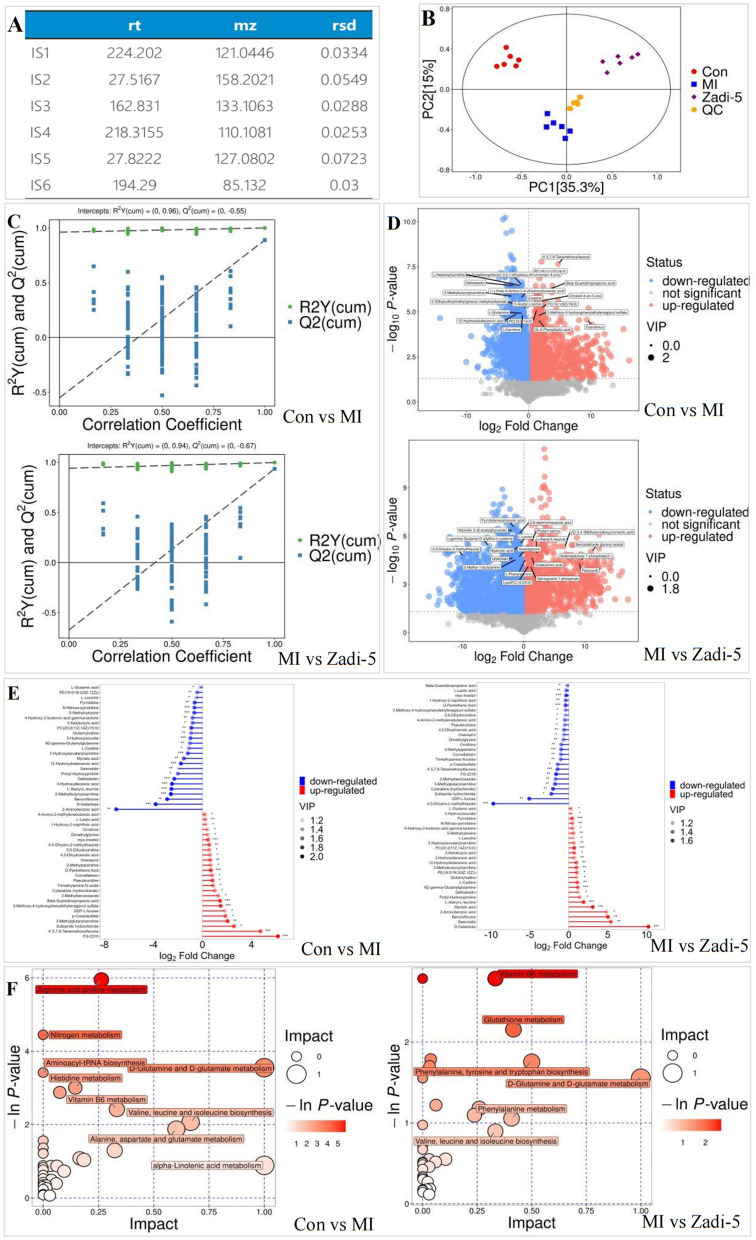
Metabolic analysis. **(A)** Response stability of the inner standard in QC samples. **(B)** PCA of Con, MI, and Zadi-5 groups. **(C)** OPLS-DA Con compared to MI and MI compared to Zadi-5. **(D)** Fire plot diagram of Con compared to MI and MI compared to Zadi-5. **(E)** Matchstick analysis of Con vs. MI group and MI vs. Zadi-5. **(F)** Metabonomic pathway analysis of Con vs. MI group, and Con vs. MI group.

The response stability of the inner standard in quality control (QC) samples was expressed by the relative standard deviation (RSD) level, which assesses the precision of data processing. Therefore, the smaller the RSD level (RSD ≤ 10%), the more stable the system and the higher the data quality. Figure 4A shows that the data quality of this experiment was very high. Principal component analysis (PCA) was used to analyze the metabolic data to characterize the overall impact of Zadi-5 on MI rats. The analysis revealed a clear separation among the groups (Figure 4B). Subsequent metabolic analyses were performed for the Con, MI, and Zadi-5 groups. Orthogonal partial least squares-discriminant analysis (OPLS-DA) showed that the metabolites of the Con vs. MI and MI vs. Zadi-5 groups clustered distinctly with Q2 (0, −0.55) vs. R2Y (0, 0.96) and Q2 (0, −0.67) vs. R2Y (0, 0.94) (Figure 4C). The volcano diagram shows the dramatic up- and downregulation of metabolites in the three groups. Three criteria were used to screen differential metabolites: (1) variable importance in projection (VIP) > 1.0, (2) *p* < 0.05, and (3) fold change (FC) > 1.2 or < 0.8 (Figure 4D). There were 105 upregulated and 127 downregulated metabolites in the MI group compared to the Con group, and 92 upregulated and 119 downregulated metabolites in the Zadi-5 group compared to the MI group. Thus, 50 disease-related metabolites were identified as key metabolic targets of Zadi-5 in MI (Figure 4E). Subsequent analyses were based on these differential metabolites.

#### 3.3.2 Metabolic signaling pathway enrichment

Fifty differential metabolites between the Con and MI groups, as well as those reversed by Zadi-5, were used to identify the metabolite pathways of Zadi-5 during MI (Figure 4F). The following metabolites were enriched in the D-glutamine–D-glutamate metabolism pathway.

### 3.4 MetOrigin tracing analysis

#### 3.4.1 Origin of differential metabolites and pathways

**Figure 5:**
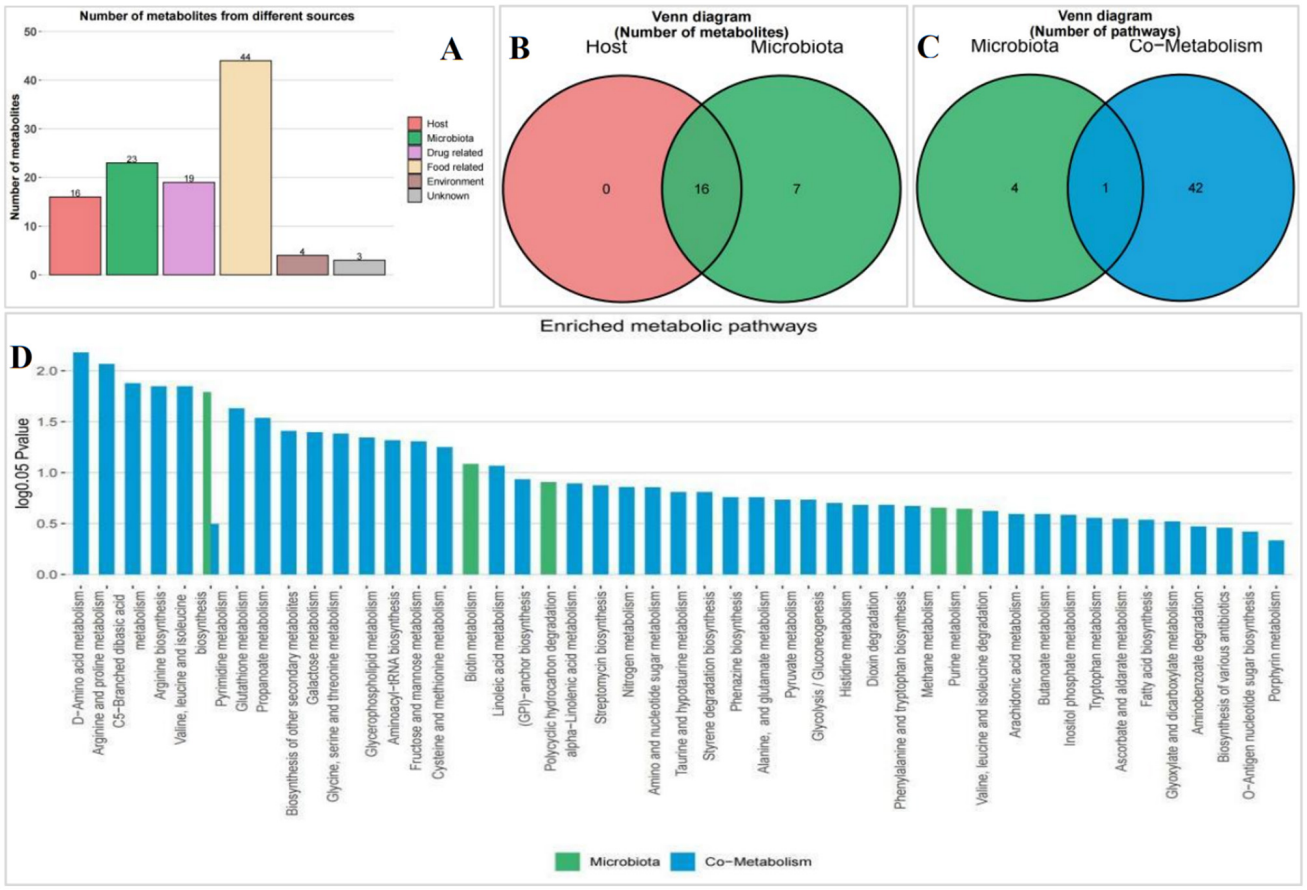
Metorigin tracing analysis of differential metabolites. **(A)** Number of different metabolites from different sources. **(B)** Venn diagram of metabolites from microbiota and host. **(C)** Venn diagram of the microbiota and co-metabolic pathways. **(D)** Enriched pathway of microbiota and co-metabolism.

MetOrigin tracing analysis was used to elucidate the origin of the identified metabolites and the functions of the metabolic pathways. Fifty differential metabolites associated with Zadi-5 under MI were identified: 16 bacteria-host, 7 bacterial-specific, 0 host-specific, 19 drug-related, 44 food-related, 4 environmental, and 1 unknown metabolite (Figures 5A, B). Metabolite pathway enrichment analysis indicated that 4 metabolic pathways were paired with the microbiota, and 42 metabolic pathways were paired with the co-metabolism metabolic pathway (Figure 5C). According to functional analysis, pyrimidine metabolism was dominant in the microbial community and enriched in the co-metabolism pathway. D-amino acid metabolism (including glutamate–glutamine) was the top pathway of co-metabolism between microbes and hosts for Zadi-5 effects in MI (log *p* > 1.5) (Figure 5D).

#### 3.4.2 Biological and statistical correlations between bacteria and metabolites using Sankey networks

**Figure 6:**
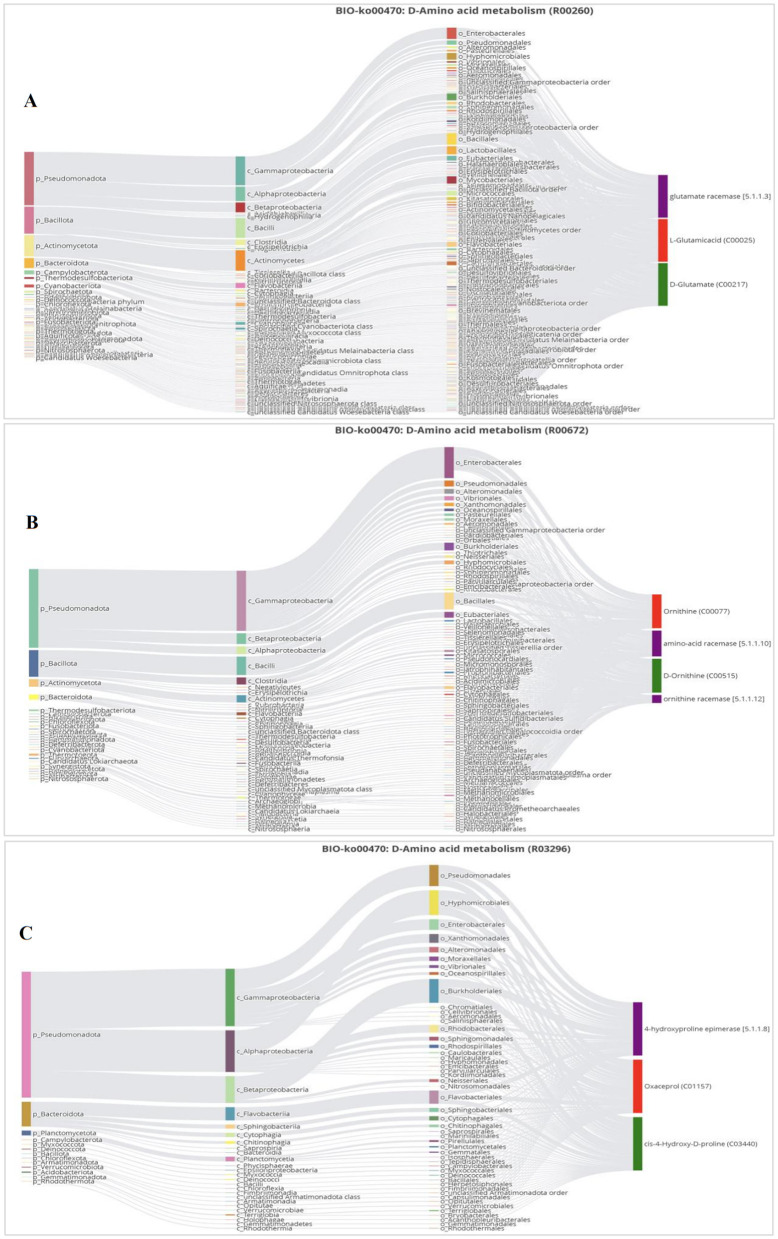
Sankey diagram of D-amino acid metabolism. **(A)** Reaction of R00260 L-glutamic acid to D-glutamate. **(B)** Reaction of R00672 ornithine to D-ornithine. **(C)** Reaction of R03296 oxaceprol to cis-4-hydroxy-D-proline.

The further visualization of statistical correlations and biological relationships between microbiota and metabolites was based on the Sankey networks of MetOrigin. D-amino acid metabolism is considered the most essential metabolic pathway associated with MI. The R00260 reaction converts L-glutamic acid to D-glutamate by glutamate racemase. The R00672 reaction converts ornithine to D-ornithine by amino-acid racemase and ornithine racemase. The R03296 reaction converts oxaceprol to cis-4-hydroxy-D-proline through 4-hydroxyproline epimerase. All these products belonged to the phyla *Pseudomadota* (*Proteobacteria*), *Bacillota (Firmicutes*), and *Actinomycetota* (*Actinobacteria*), which include the classes *Gammaproteobacteria, Alphaproteobacteria, Betaproteobacteria, Bacilli*, and *Actinomycetes* (Figures 6A–C).

### 3.5 Relative abundance of intestinal microbiota related to the metabolism

**Figure 7:**
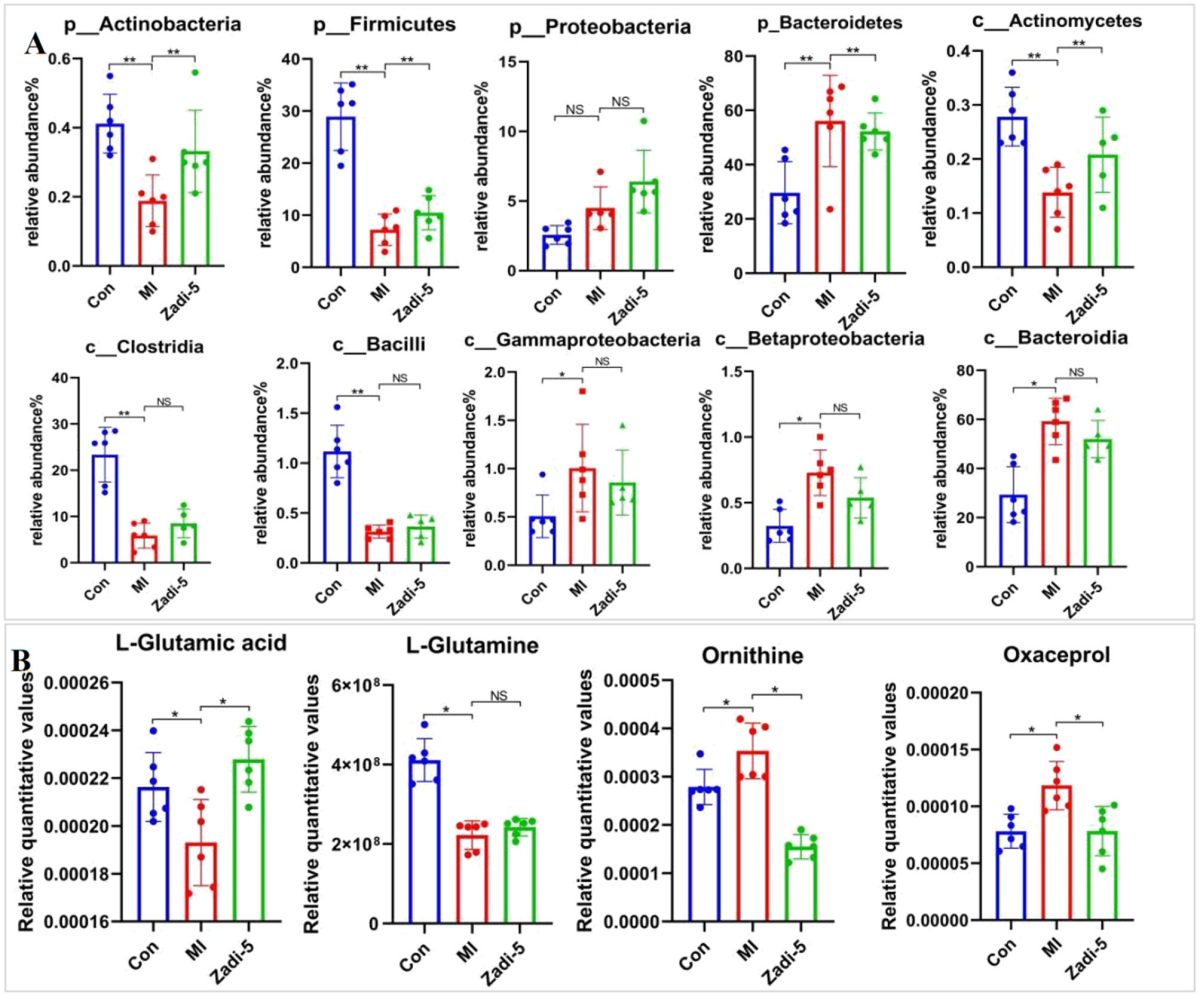
Relative levels of intestinal microbiota in feces and metabolites in serum. **(A)** Relative abundance of intestinal microbiota at the phylum and class levels. **(B)** Relative level of metabolomics in the glutamine-glutamate metabolic pathway. *P < 0.05, **P < 0.01, NS represents no statistical significance.

At the phylum taxon, the relative abundance of *Actinobacteria* and *Firmicutes* was decreased (*p* < 0.01), and that of *Bacteroidetes* and *Proteobacteroidetes* in the MI group was increased compared with the Con group. Compared with the MI group, Zadi-5 regulated the relative abundance of *Actinobacteria, Firmicutes*, and *Bacteroidetes*. In the class taxon, the MI group showed a significantly increased abundance of *Gammaproteobacteria, Betaproteobacteria*, and *Bacteroidia* and a decreased abundance of *Actinomycetes, Clostridia*, and *Bacilli*. Compared with the MI group, Zadi-5 showed regulatory effects to some degree (Figure 7A).

### 3.6 Relative quantitative values of metabolites related to glutamate–glutamine metabolism

The metabolites of glutamate–glutamine metabolism are illustrated to display the features of the relative quantitative value distribution in the Con, MI, and Zadi-5 groups. Compared to the Con group, the relative quantitative values of L-glutamic acid and L-glutamine in the MI group were decreased and regulated in the Zadi-5 group. Compared to the Con group, the relative quantitative values of ornithine and oxaceprol were increased in the MI group and regulated by Zadi-5 (Figure 7B).

## 4 Discussion

Zadi-5 is a traditional Mongolian medicine used to treat cardiac disorders, such as palpitations, sychnosphygmia, and angina symptoms, with definite results (Xiaoqin et al., 2022; Erdunni, 2024). Clinically, coronary atherosclerosis–induced MI is the most common form of atherosclerosis, which is closely related to dietary habits. High-fat, high-sugar, and high-calorie diets, as well as unhealthy lifestyle habits, such as smoking and drinking, can cause gut microbiota disorders or their metabolites. This study established animal models of MI using HFD and ISO injections. ISO is a non-selective β-adrenoceptor agonist that causes cardiac overload and energy depletion, which induces significant myocardial ischemic damage (Adameova et al., 2009; Kim et al., 2025). Our model is recognized as a commonly used MI rat model (Hareeri et al., 2023). ISO administration in experimental rodents shows high reproducibility, validity, and low mortality compared to other models. It is a simple, quick, and non-invasive protocol that produces myocardial injury similar to that observed in humans (Xing et al., 2022). Due to such similarity of this model, ISO is widely used for the evaluation of cardioprotective effects of various drugs against MI (Mnafgui et al., 2016). Long-term HFD intake leads to lipid metabolism disorders, which induce coronary stenosis. Adding ISO induces myocardial ischemia, which is highly similar to human ischemic heart disease in terms of pathogenesis. The results of this experiment indicated that in the MI group, the ST segment was significantly depressed, serum LDH, CTn-T, CK-MB, TC, TG, and LDL-C levels were increased, and cardiac ischemic histopathological changes were more prominent than those in the Con group. However, both high and low Zadi-5 doses protected the subjects from myocardial injury in terms of the aforementioned markers in this model. Compared with the Con group, HDL-C was increased in the MI group. This might be related to the self-regulation capability in rats, since HDL-C remained increased in the high- and low-dose Zadi-5 groups. In this study, Zadi-5 cardioprotective effects were not dose-dependent within the tested range. As the low-dosage group was equivalent to the standard clinical medication dose, this dose was used to further perform metabolomics and metagenomics research to investigate the myocardium-protecting mechanism of Zadi-5.

The gut-heart axis is an emerging area of research with the potential to provide new insights into CVD pathophysiology and uncover innovative therapeutic targets. The gut-heart axis represents an emerging frontier in understanding the intricate interplay between gut microbiota, metabolic products, and cardiovascular health. A healthy gut microbiota structure and metabolite function are essential for maintaining healthy cardiovascular homeostasis (Cui et al., 2023; Tang et al., 2019). They represent a unique ecosystem responsible for the host's physiological activities, such as metabolism, immune regulation, nourishment synthesis, and digestion. The gut microbiota can influence the heart by producing bioactive compounds that cross the gut epithelial barrier, enter systemic circulation, and directly affect cardiac tissues (Snelson et al., 2025; Epelde, 2025). Healthy microbiota mainly comprises phyla of *Firmicutes* and *Bacteroidetes*, which account for 70%−90% of microorganisms. *Actinobacteria* are important for the preservation of gut homeostasis by maintaining intestinal barrier function, as well as regulating mucin biosynthesis and catabolism (Epelde, 2025). Notably, differences in gut microbiota composition were observed between individuals with and without heart disease, suggesting a potential link between microbial perturbations and cardiac dysfunction. A clinical study showed that *Firmicutes* and *Bacteroidetes* were the two most abundant phyla; furthermore, at the class level, *Actinobacteria, Bacteroidia, Bacilli*, and *Clostridia* were decreased in patients with coronary artery disease and non-alcoholic fatty liver disease compared with healthy controls (Hu et al., 2022). Thus, the gut microbiota plays a crucial role in the production of various metabolites. The host metabolites undergo modification by gut bacteria, which also synthesize *de novo* metabolites. In this experiment, fecal metagenomic analysis showed that the diversity and abundance of the gut microbiota in MI model rats were disordered. Zadi-5 regulates alpha and beta diversities. Regarding microbiota abundance, Zadi-5 regulated the relative abundance of *Actinobacteria, Bacteroidetes*, and *Firmicutes* in the phylum taxonomy. In class taxonomy, Zadi-5 regulates the abundance of *Gammaproteobacteria* and *Bacteroidia* and decreases the abundance of *Clostridia, Bacilli*, and *Erysipelotrichia*. According to the KEGG pathway enrichment analysis, metabolism was the dominant level 1 pathway, with energy metabolism and amino acid metabolism being enriched. Thus, blood serum metabolomic analysis was performed to comprehensively understand the potential protective mechanisms of Zadi-5 in MI. In recent years, metabolomics has been widely used to explore the efficacy and mechanism of traditional Chinese medicine (Feng et al., 2023). MI is characterized by an imbalance between myocardial blood oxygen supply and demand, which causes cardiac metabolic abnormalities. The reliance of the ischemic heart on amino acids as a fuel source may increase during the shortage of cardiomyocyte energy (Tang, 2023). Amino acids such as glutamic acid (Glu), arginine, ornithine, and glutamine (Gln) can enter the Krebs cycle as alpha-ketoglutarate (A-KG) or via the glutamate-ornithine-proline pathway to provide energy. Gln is primarily synthesized from L-glutamate and ammonia by Gln synthetases and is hydrolyzed to glutamate. Extracellular Gln is transported into cells via the cystine/glutamate antiporter system and then catabolized by glutaminase in the mitochondria into Glu. Then, Glu can be converted into A-KG by Glu dehydrogenase in the mitochondria or by aminotransferases in either the cytosol or mitochondria. AKG can be used to generate adenosine triphosphate (ATP) via the tricarboxylic acid cycle or for the synthesis of nucleotides, proteins, and lipids. Afterward, cytosolic Glu is then used to synthesize glutathione, an antioxidant that protects cells from oxidative stress. Glu plays an important role in both glutathione and Gln–Glu metabolisms. Therefore, improving the antioxidant capacity of the heart by enhancing the production of glutathione may protect against apoptosis and stress tolerance by elevating heat shock proteins and stabilizing epithelial inflammatory pathways. When MI occurs, Gln–Glu levels decrease in the blood serum and cardiomyocytes, Glu is exhausted, and ornithine is released into the blood serum. This was observed in our metabolomic results, showing that L-Glu in the MI group was decreased, whereas ornithine and oxaceprol were increased, which was regulated by Zadi-5. This analysis revealed that Gln–Glu metabolism was the most significant pathway investigated in this study. This pathway belongs to D-amino acid metabolism and is the key co-metabolism pathway that most previous investigations have focused on in terms of metabolic dysregulation in heart diseases.

Metorigin is a public, visualized, interactive website, which can be used to explore the complex relationships between microbiota and metabolomics based on different sources of metabolite enrichment analysis and the relevant microbiota Sankey network. D-amino acid metabolism mainly originates from co-metabolism, which is used for peptidoglycan formation, biofilm dispersion, and adaptation to environmental changes. Additionally, various enzymes, including amino acid racemases, catalyze the isomerization of D-amino acids and L-amino acids in bacteria (Miyamoto and Homma, 2021). According to the Metorigin analysis in this study, Glu-Gln metabolism was associated with the phylum taxon of *Actinobacteria, Firmicutes, Bacteroidetes*, and *Proteobacteroidetes* and the class taxon of *Gammaproteobacteria, Betaproteobacteria, Bacteroidia, Actinomycetes, Clostridia*, and *Bacilli*. In summary, this study demonstrated the myocardium-protecting effects of Zadi-5 and further illustrated the protective mechanisms of Zadi-5 for improving the structure and function of gut microbiota to regulate the Glu-Gln metabolism pathway.

Mongolian medicine has a history of more than 2,000 years, forming a complete theoretical system to provide a unique insight into human life activities and rich drug resources. The formula of Zadi-5, a digestive functional and cardioprotective dual-function medication, has been used to treat CVD for a long time. A previous study illustrated that Zadi-5 has cardioprotective effects; however, the mechanisms of MI treatment through the gut-heart axis have not been clarified. According to the Mongolian medicine theory, the heart and intestine have a subordinate relationship, considering that the pathology and physiology are closely related. This study, guided by the Mongolian medicine theory, not only revealed the cardioprotective effect of Zadi-5 from the perspective of biomedicine but also provided a scientific basis for the intrinsic connection between the intestine and heart in Mongolian medicine, enriching Mongolian medicine. Zadi-5, a traditional formula characterized by a multi-component, multi-target synergistic reaction, participates in gut microbiota host co-metabolism to regulate the Glu-Gln metabolic pathway. This may represent one of the multiple complicated protective mechanisms of Zadi-5 against MI.

This study has some limitations. First, an MI animal model was established using HFD and ISO injection. The lack of a fecal translocation experiment limited our ability to define the specific role of the microbiota in the protective effects of Zadi-5 in MI rats. Second, as it is limited by an untargeted metabolomic test in blood serum, we plan to quantify the metabolite levels using LC-MS/MS and NMR techniques in the next study. Finally, using gene knockout or inhibitor methods to explore effective pathways *in vitro* would be informative. Directed by the complicated chemical components of Zadi-5, this study could not completely illustrate the protective mechanisms in MI injury. In the future, providing evidence for its wide use in clinical and scientific research is anticipated based on the Mongolian medical theory connected to interdisciplinary research to explore the cardioprotective mechanisms of Zadi-5 more completely.

## Data Availability

The data presented in this study are publicly available. The data can be found here: https://www.ebi.ac.uk/metabolights/MTBLS13780.

## References

[B1] Adameova A. Xu Y. J. Duhamel T. A. Tappia P. S. Shan L. Dhalla N. S. (2009). Anti-atherosclerotic molecules targeting oxidative stress and inflammation. Curr. Pharmaceut. Design 15, 3094–3107. doi: 10.2174/138161209789058048 19754384

[B2] Alshehri M. A. (2022). Cardioprotective properties of Artemisia herba alba nanoparticles against heart attack in rats: a study of the antioxidant and hypolipidemic activities. Saudi J. Biol. Sci. 29, 2336–2347. doi: 10.1016/j.sjbs.2021.12.009 35531258 PMC9072917

[B3] Ashokkumar K. Simal-Gandara J. Murugan M. Dhanya M. K. Pandian A. (2022). Nutmeg (*Myristica fragrans* Houtt.) essential oil: a review on its composition, biological, and pharmacological activities. Phytother. Res. 36, 2839–2851. doi: 10.1002/ptr.7491 35567294 PMC9541156

[B4] Biswas P. Ghorai M. Mishra T. Gopalakrishnan A. V. Roy D. Mane A. B. . (2022). *Piper longum* L.: a comprehensive review on traditional uses, phytochemistry, pharmacology, and health-promoting activities. Phytother. Res. 36, 4425–4476. doi: 10.1002/ptr.7649 36256521

[B5] Buza V. Niculae M. Hanganu D. Pall E. Burtescu R. F. Olah N. K. . (2022). Biological activities and chemical profile of *Gentiana asclepiadea* and *Inula helenium* ethanolic extracts. Molecules 27:3560. doi: 10.3390/molecules27113560 35684497 PMC9182457

[B6] Chen B. Patel S. Bao L. Nadeem D. Krittanawong C. (2024). Pro-inflammatory food, gut microbiota, and cardiovascular and pancreatic diseases. Biomolecules 14:210. doi: 10.3390/biom14020210 38397447 PMC10886602

[B7] Cui H. Han S. Dai Y. Xie W. Zheng R. Sun Y. . (2023). Gut microbiota and integrative traditional Chinese and western medicine in prevention and treatment of heart failure. Phytomedicine 117:154885. doi: 10.1016/j.phymed.2023.154885 37302262

[B8] Datta S. Pasham S. Inavolu S. Boini K. M. Koka S. (2024). Role of gut microbial metabolites in cardiovascular diseases-current insights and the road ahead. Int. J. Mol. Sci. 25:10208. doi: 10.3390/ijms251810208 39337693 PMC11432476

[B9] Epelde F. (2025). The role of the gut microbiota in heart failure: pathophysiological insights and future perspectives. Medicina (Kaunas) 61:720. doi: 10.3390/medicina61040720 40283011 PMC12028989

[B10] Erdunni H. (2024). Effect analysis of Mongolian medicine Zadi-5, Xiaoyao Powder combined with psychotherapy in the treatment of post-stroke depression. China J. Ethnic Med. 30, 25–27. doi: 10.16041/j.cnki.cn15-1175.2024.06.016

[B11] Feng L. Lisha A. Li H. Mu X. Ta N. Bai L. . (2023). Pharmacological mechanism of *Aucklandiae radix* against gastric ulcer based on network pharmacology and *in vivo* experiment. Medicina (Kaunas). 59, 666. doi: 10.3390/medicina59040666 37109624 PMC10140907

[B12] Goldsborough E. III. Tasdighi E. Blaha M. J. (2023). Assessment of cardiovascular disease risk: a 2023 update. Curr. Opin. Lipidol. 34, 162–173. doi: 10.1097/MOL.0000000000000887 37431303

[B13] Guo J. Xu F. Ji H. Jing Y. Shen L. Weng X. . (2022). isolevuglandins scavenger ameliorates myocardial ischemic injury by suppressing oxidative stress, apoptosis, and inflammation. Front. Cell. Dev. Biol. 10:836035. doi: 10.3389/fcell.2022.836035 35356291 PMC8959416

[B14] Hareeri R. H. Alam A. M. Bagher A. M. Alamoudi A. J. Aldurdunji M. M. Shaik R. A. . (2023). Protective effects of 2-methoxyestradiol on acute isoproterenol-induced cardiac injury in rats. Saudi Pharm. J. 31:101787. doi: 10.1016/j.jsps.2023.101787 37766820 PMC10520946

[B15] Hu X. Zhou R. Li H. . (2022). Alterations of gut microbiome and serum metabolome in coronary artery disease patients complicated with non-alcoholic fatty liver disease are associated with adverse cardiovascular outcomes. Front. Cardiovasc. Med. 8:805812. doi: 10.3389/fcvm.2021.805812 35047580 PMC8761954

[B16] Jain H. Marsool M. D. M. Goyal A. Sulaiman S. A. Fatima L. Idrees M. . (2024). Unveiling the relationship between gut microbiota and heart failure: recent understandings and insights. Curr. Probl. Cardiol. 49(1 Pt C):102179. doi: 10.1016/j.cpcardiol.2023.102179 37923029

[B17] Kim S. H. Kee H. J. Zhou H. Park H. Lee S. H. Sim D. S. . (2025). Chlorogenic acid attenuates cardiac hypertrophy and fibrosis by downregulating galectin 3. Sci. Rep. 15:26925. doi: 10.1038/s41598-025-12222-0 40707644 PMC12290003

[B18] Ko S. H. Kim H. S. (2020). Menopause-associated lipid metabolic disorders and foods beneficial for postmenopausal women. Nutrients 12:202. doi: 10.3390/nu12010202 31941004 PMC7019719

[B19] Kondapalli N. Katari V. Dalal K. K. Paruchuri S. Thodeti C. K. (2025). Microbiota in Gut-heart axis: metabolites and mechanisms in cardiovascular disease. Compr. Physiol. 15:e70024. doi: 10.1002/cph4.70024 40542540 PMC12181760

[B20] Miyamoto T. Homma H. (2021). D-Amino acid metabolism in bacteria. J. Biochem. 170, 5–13. doi: 10.1093/jb/mvab043 33788945

[B21] Mnafgui K. Hajji R. Derbali F. Khlif I. Kraiem F. Ellefi H. . (2016). Protective effect of hydroxytyrosol against cardiac remodeling after isoproterenol-induced myocardial infarction in rat. Cardiovasc. Toxicol. 16, 147–155. doi: 10.1007/s12012-015-9323-1 25846342

[B22] Mokhtari M. Khalil D. Farzadfar F. Daroudi R. Asadi-Lari M. (2022). The burden of cardiovascular disease attributable to modifiable risk factors and cost-effectiveness analysis of IraPEN program in the general population of Iran. Med. J. Islam. Repub. Iran 36:73. doi: 10.47176/mjiri.36.73 36128278 PMC9448466

[B23] Nageswaran V. Carreras A. Reinshagen L. Beck K R. Steinfeldt J. Henricsson M. . (2025). Gut microbial metabolite imidazole propionate impairs endothelial cell function and promotes the development of atherosclerosis. Arterioscler. Thromb. Vasc. Biol. 45, 823–839. doi: 10.1161/ATVBAHA.124.322346 40143816 PMC12017598

[B24] Pitt B. Diez J. (2024). Possible Role of Gut Microbiota Alterations in Myocardial Fibrosis and Burden of Heart Failure in Hypertensive Heart Disease. Hypertension 81, 1467–1476. doi: 10.1161/HYPERTENSIONAHA.124.23089 38716665

[B25] Snelson M. Muralitharan R. Liu C. F. Markó L. Forslund S. K. Marques F. Z. . (2025). Gut-heart axis: the role of gut microbiota and metabolites in heart failure. Circ. Res. 136, 1382–1406. doi: 10.1161/CIRCRESAHA.125.325516 40403109 PMC12101525

[B26] Tang W. H. W. (2023). Dysregulated amino acid metabolism in heart failure: role of gut microbiome. Curr. Opin. Clin. Nutr. Metab. Care 26, 195–200. doi: 10.1097/MCO.0000000000000897 36729604

[B27] Tang W. H. W. Li D. Y. Hazen S. L. (2019). Dietary metabolism, the gut microbiome, and heart failure. Nat. Rev. Cardiol. 16, 137–154. doi: 10.1038/s41569-018-0108-7 30410105 PMC6377322

[B28] Tiwari B. R. Naseeruddin Inamdar M. Orfali R. Alshehri A. Alghamdi A. Almadani M. E. . (2023). Comparative evaluation of the potential anti-spasmodic activity of *Piper longum, Piper nigrum, Terminalia bellerica, Terminalia chebula*, and *Zingiber officinale* in experimental animals. Saudi Pharm J. 31:101705. doi: 10.1016/j.jsps.2023.101705 37576742 PMC10413155

[B29] Wei L. Su S. Sun Y. Qi L. Hu R. (2021). Study on the cardiomyocyte protective effect of Zaidi-5wei pills in rats with myocardial ischemia-reperfusion injury model. Chin. J. Natl. Med. 27, 50–53. doi: 10.16041/j.cnki.cn15-1175.2021.12.024

[B30] Wuri G. Weilisi W. L. G. Sus A. Tong L. Bu T. Hu R. (2024). The mechanism of action and validation of zadi-5 pills in improving myocardial ischemia reperfusion injury. China Pharmacy 35, 442–448.

[B31] Xiaoqin N. Dongxue C. Pai L. Zhanhong Q. (2022). Modern research progress on Mongolian medicine Roukou Wuwei Pill (Zadi-5). Jilin J. Tradit. Chinese Med. 42, 221–224. doi: 10.13463/j.cnki.jlzyy.2022.02.025

[B32] Xing Z. Yang C. He J. Feng Y. Li X. Peng C. . (2022). cardioprotective effects of aconite in isoproterenol-induced myocardial infarction in rats. Oxid. Med. Cell. Longev. 2022:1090893. doi: 10.1155/2022/1090893 36600948 PMC9807305

[B33] Zhang X. Zhao Y. Zhao X. Zhang J. Diao J. Jia S. . (2023). Anti-inflammatory, cardioprotective effect of gypenoside against isoproterenol-induced cardiac remodeling in rats via alteration of inflammation and gut microbiota. Inflammopharmacology 31, 2731–2750. doi: 10.1007/s10787-023-01307-9 37603159

