# Supplementary material for: Metagenomics and metabolomics integrated to explore the protective mechanisms of Mongolian medicine Zadi-5 in myocardial ischemic model rats
Source: Front Microbiol. 2026 Feb 9;16:1677322. doi: 10.3389/fmicb.2025.1677322 (PMC12926489; doi:10.3389/fmicb.2025.1677322)

**1. Sample preparing**

200 mg of Zadi-5 powder add 1000 µL of 80% methanol solution and grinding beads, grind 5

min, vortex for 10 min. Centrifuge at 4℃ for 10 min with a centrifugal force of 20,000 xg, and take the supernatant for analysis on the computer.

**2. Detection conditions**

**2.1Mass spectrometry conditions**

Ion source: Electrospray ionization source (ESI)

Scanning mode: positive and negative ion switching scan

Detection method: Full mass/dd-MS2

Resolution: 70000 (full mass); 17500 (dd-MS2)

Scan range: 100.0～1500.0 m/z

Spary Voltage: 3.8 kV (Positive)

Capillary Temperature: 300 ℃

Collision gas: high-purity argon (purity ≥99.999 %)

Collision energy (N) CE: 30

Sheath gas: nitrogen (purity ≥99.999%), 40 Arb

Auxiliary gas: nitrogen (purity ≥99.999%), 15 Arb, 350 ℃

Data collection time: 30.0 min

**2.2 Chromatographic conditions**

Column: AQ-C18, 150×2.1mm, 1.8 µm, Welch

Flow rate: 0.30 mL/min

Aqueous phase: 0.1% formic acid in water

Organic phase: methanol

Needle wash: methanol

Column oven temperature: 35°C

Autosampler temperature: 10.0°C

Autosampler injection volume: 5.00 µL

Table1 Chromatographic gradients

| Time (min） | Water phase ratio (%) | organic phase ratio(%) |
| --- | --- | --- |
| 1 | 98 | 2 |
| 5 | 80 | 20 |
| 10 | 50 | 50 |
| 15 | 20 | 80 |
| 20 | 5 | 95 |
| 25 | 5 | 95 |
| 26 | 98 | 2 |
| 30 | 98 | 2 |

3.3 Data Analysis

The data collected by high-resolution liquid mass was completed by CD2.1 (Thermo Fisher).

Process the database search and comparison (mzCloud) ，After preliminary sorting.


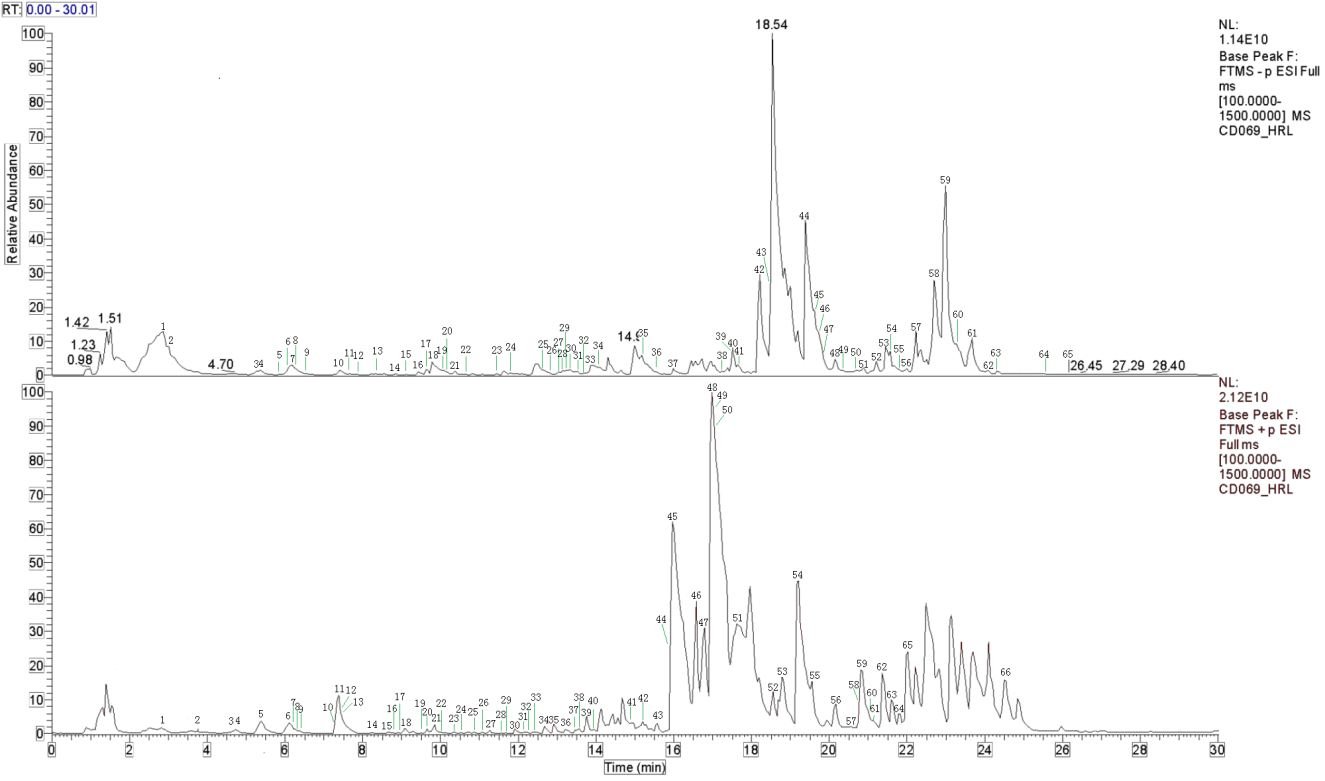

Supplement: Supplementary file 1 [file Supplementary_file_1.docx]
